# Low-Overhead Accrual Failure Detector

**DOI:** 10.3390/s120505815

**Published:** 2012-05-04

**Authors:** Xiao Ren, Jian Dong, Hongwei Liu, Yang Li, Xiaozong Yang

**Affiliations:** School of Computer Science and Technology, Harbin Institute of Technology, Harbin 150001, China

**Keywords:** failure detection, accrual failure detector, adaptive

## Abstract

Failure detectors are one of the fundamental components for building a distributed system with high availability. In order to maintain the efficiency and scalability of failure detection in a complicated large-scale distributed system, accrual failure detectors that can adapt to multiple applications have been studied extensively. In this paper, an new accrual failure detector—LA-FD with low system overhead has been proposed specifically for current mobile network equipment on the Internet whose processing power, memory space and power supply are all constrained. It does not rely on the probability distribution of message transmission time, or on the maintenance of a history message window. By simple calculation, LA-FD provides adaptive failure detection service with high accuracy to multiple upper applications. The related experiments and results have also been presented.

## 1. Introduction

Failure detector is one of the fundamental components for building a distributed system with high availability [1]. By providing the processes' failure information to the system, it supports the solution of many basic issues (such as consensus and atomic broadcasting, *etc.*) in an asynchronous system. Failure detection was proposed and formally defined by Chandra and Toueg [2] as an effective way to enhance the asynchronous system computational model. With the increasing demands on capability in distributed systems, failure detectors have been widely applied to many fields including grid computing [3], cluster management [4] and peer-to-peer networks [5]. As a fundamental component, more and more challenges to the efficiency and scalability [6] of failure detectors have been posed by the expanding system scale and increasingly complex distributed applications. How to achieve good detection speed and accuracy with low detection load has become a hot research topic in this field.

Adaptive failure detectors have been proposed as an important approach to solve this problem. They adjusts the detector's parameters automatically so that the system's requirement on the indicator of effectiveness can be met with low load under different network environments. Chen [7] and Bertier [8] proposed a series of QoS-based adaptive failure detection algorithms based on a probability network model. These algorithms have achieved adaptive adjustment in the quantitative control of detector parameters and greatly improved the detector's control accuracy and effectively reduced detection load. However, with the development of various network applications, multiple applications are often running simultaneously in large-scale systems such as grid, P2P and cloud computing. They have different failure detection QoS requirements. Taking into account the impact of load on scalability, we can't supply separate failure detectors for each application. Therefore, here comes another requirement for adaptive failure detectors, that is, that they can adapt to different QoS requirements demanded by multiple applications. This has become an important issue in the research of failure detection in large-scale distributed systems [6].

Hayashibara [9] first launched the research in this area and proposed the concept of accrual detector. It allows a complete decoupling between monitoring and interpretation in traditional models of failure detection. By outputting a continuous value associated with the status of a process rather than a binary value simply representing success or failure, upper applications can interpret detection results according to their own QoS requirements. Therefore, multiple applications can share the same detector and the failure detection load can be effectively reduced in large-scale distributed systems. Currently many implementations of accrual detectors have been proposed and applied satisfactorily to some well-known systems, such as Facebook [10]. However, with the development of applications in the Internet of Things and cloud computing, network access equipment has become diversified. Mobile terminals like cell phones and tablet PCs are being used more widely. The majority of such equipment are embedded systems whose processing power, memory space and power supply are all constrained, but the previously proposed accrual detectors require the probability distribution model for message transmission delay. For example, the *ϕ*-detector uses normal distribution [11], Cassandra uses exponential distribution [10], and Benjamin uses gamma distribution [12]. Furthermore, those detectors need a certain memory space to save a large history message window. At each detection cycle, a large amount of calculation is needed to compute the probability distribution parameters and detector parameters. For most mobile terminals, these system overheads for failure detection have an important impact on system performance and battery consumption, and regarding failure detection itself, Gillen [13] has pointed out that the transmission delays caused by performance degradation would also have great impact on detection accuracy.

Therefore, aiming at mobile devices with constrained resource, we have proposed an accrual failure detector with low system overhead. It does not rely on the probability distribution of message transmission delay, or on the maintenance of history message windows. Through simple calculations, it is able to provide an adaptive failure detection service with high accuracy to multiple upper applications.

## 2. Algorithm Description

### 2.1. System Model

We consider an asynchronous distributed system consisting of *n* processes, *∏* = {*p_1_*, *p_2_*, …, *p_n_*}. Because the failure detector is running as a basic component in the node, one simple topology is considered, and we assume that each pair of processes is connected by a communication channel that can be used to send and receive messages. The type of failure is crash and channels are fair-lossy channels. No synchronized clock is assumed.

### 2.2. Basic Failure Detection Strategy

**Figure 1.**
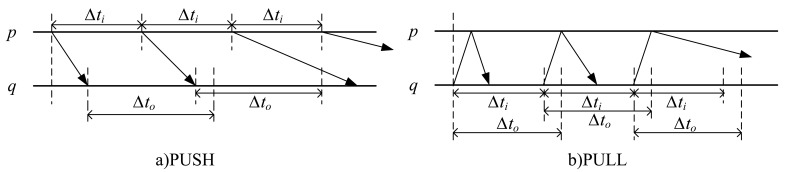
Heartbeat detection approaches.

Heartbeat is a common method to implement failure detectors. The detection modules detect each other's status by sending heartbeat messages periodically at duration Δ*t_i_*. According to the different modes of implementation, there are two monitoring approaches: PUSH and PULL. For two processes *p* and *q* in system, where *q* is monitoring *p*, the two basic approaches are described in Figure 1.

Both of the approaches detect each other's status by sending out heartbeat messages periodically at duration Δ*t_i_*. The difference is, in PUSH, the monitored process *p* initiatively sends a periodical message “I am alive” to process *q*, informing *q* that *p* is still alive; while in PULL, process *q* sends a probing message “Are you alive?” to the monitored process *p* periodically. After receiving the query message, the monitored process *p* passively replies an “I am alive!” message to indicate its status. For traditional failure detectors based on timeout mechanism, an appropriate time-out value Δ*t_o_* needs to be set. If no response message is received after Δ*t_o_*, the monitored process will be suspected as a failure. Obviously, the PULL approach needs twice the number of messages to achieve the same performance, but this does not affect its scalability. However, PULL is an initiative detection method which launches detection only when needed, and it does not need the assumption of a global synchronization clock. This is very important for current complicate large-scale distributed applications. Therefore, PULL employed as the basic detection strategy in this paper.

### 2.3. Basic Idea of the Algorithm

One of the key factors that affect the performance of an accrual failure detector is the calculation method for *sl*(*t*). Whether the value of *sl*(*t*) can give an accurate description about the actual failure status of a process determines the detector's detection accuracy and delay, *etc*. In current implementations of the accrual failure detector, in order to improve the calculation precision for *sl*(*t*), we usually have to rely on the prediction of the arrival time of detection messages. An accurate prediction model will greatly increase the detector performance. Some examples of the estimation methods which are used most frequently are: estimating the arrival time of detection messages using the distribution probability of message delay, predicting possible transmission delay by a linear process based on learning, *etc*. These methods not only cause heavy computing and storage overhead but also are limited to specific distributed systems. For example, Avinash's prediction method based on exponential distribution is proposed according to the particular characteristics of the Facebook system. In order to find a prediction method with less overhead and better adaptability, we have observed transmission delays under two typical network conditions. The detection processes used in the experiment are located in Harbin, and the monitored processes are located in Beijing (China) and Pittsburgh (PA, USA) respectively. These two sets of experiments correspond to good (dataset 1 with an average delay of 82.1 ms) and poor (dataset 2 with an average delay of 1,297.8 ms) network conditions, respectively. We have observed for 24 h, respectively, and the results are shown in the figure below.

**Figure 2.**
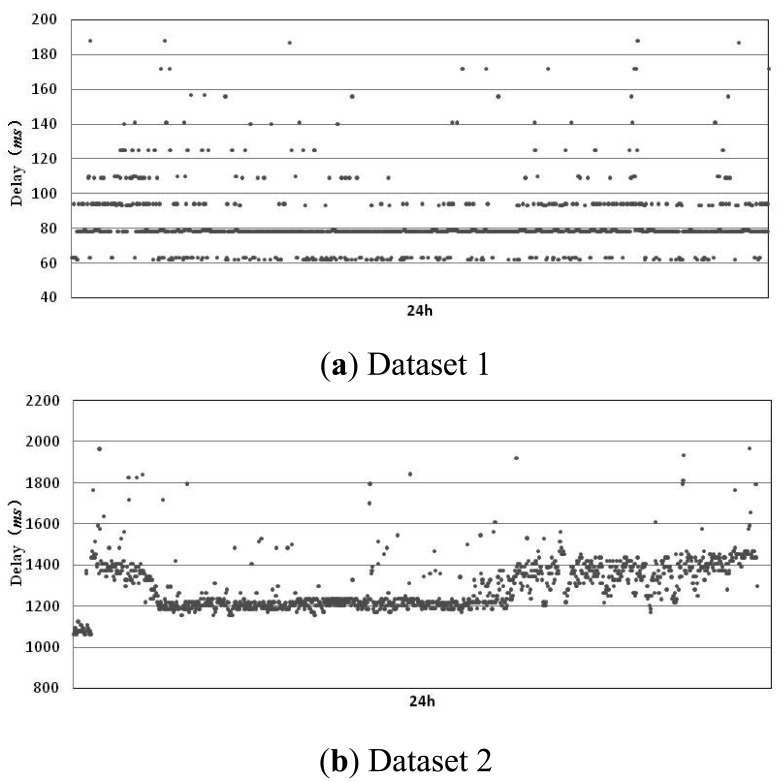
Experimental results for transmission delay.

From Figure 2, we can see that in the two different network environments, transmission delay shows a continuity (in Figure 2(a), data is centralized on 50, 80 and 100 ms, and in Figure 2(b), data is centralized on 1,200 and 1,400 ms). Only a very small number of detection messages have a large deviated transmission delay due to network congestion or message loss, *etc*. Furthermore, from the statistical data in Figure 2(a), we can get:

(1) $$P_{0}[({\text{delay}}_{i}-{\text{delay}}_{i-1})\leq 0]=73.1\%,i>1$$

Even in Figure 2(b) for a poor network environment, has also reached 56.3%. Therefore, the transmission time *delay_i_* for most detection messages is less than or close to the transmission time of previous message *delay_i_*_-1_. *delay_i_*_-1_ can be used as the predicted value for *delay_i_* to support failure detection, which means the predicted value of the *i-th* detection message is *prek_i_* = *delay_i_*_-1_. This method does not cause overhead for modeling and recording a large amount of historical data, and it's adaptive to different network environments. However, we can see from *P*_0_ that the accuracy of this method is not high, especially for the case of a poor network environment. Therefore, we refer to the evaluation method proposed by Jacobson [14] and add consideration of a safety margin to the predicted value:

(2) $${\text{margin}}_{i}={\text{margin}}_{i-1}+\alpha (\left|{\text{prek}}_{i-1}-{\text{delay}}_{i-1}\right|-{\text{margin}}_{i-1}),i>1$$

Let *α* = 0.25, for data in Figure 2(a), we have *P_m_*[*delay_i_* ≤ *delay_i_*_-1_ + *margin_i_*_-1_] = 98.9%. For Figure 2(b), *P_m_* has also reached 98.4%. Therefore, this new prediction method has greatly improved the prediction accuracy and met the needs for most failure detections. Based on this method, we have proposed the LA-FD failure detector.

### 2.4. LA-FD Failure Detector

**Figure 3.**
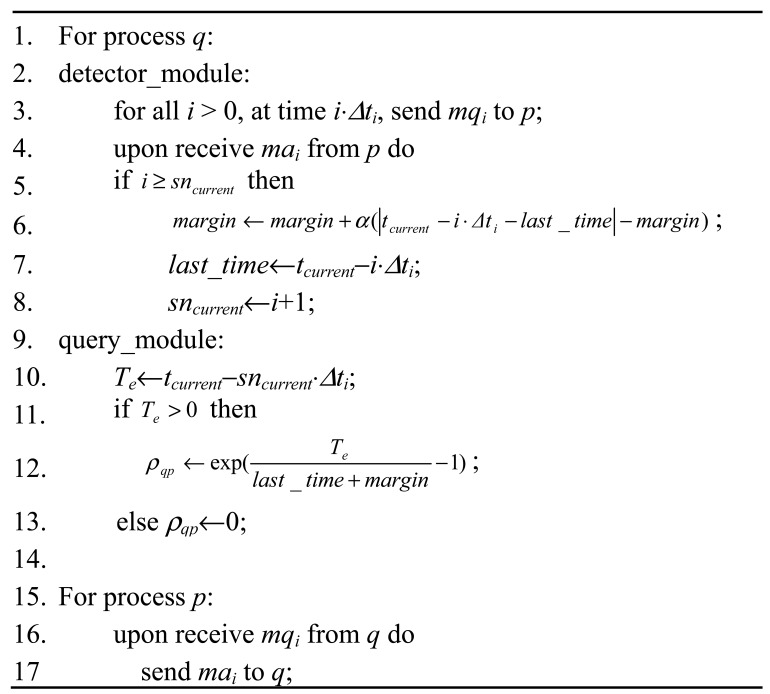
LA-FD failure detector.

LA-FD employs the PULL approach as the basic failure detection strategy. To simply the description, suppose the system consists of only two processes *p* and *q*, where *q* is monitoring *p*. The detection algorithm is shown in Figure 3.

Figure 3 shows that the LA-FD failure detector consists of a detection module and a query module. The detection module located on process *q* sends probing message *mq_i_* to the monitored process *q* at interval *Δt_i_*. After receiving the probing message *mq_i_*, process *p* immediately replies an acknowledge message *ma_i_* to indicate its status. Everytime after receiving the acknowledge message *ma_i_*, process *q* needs to calculate the margin for the next detection message and records the transmission time of current detection message. When an upper application queries the detector, it will reply with a value of *&rho:_qp_*. Then the upper application will set a threshold value *P* according to its own requirement for detection accuracy. When *&rho:_qp_* > *P*, process *p* is suspected as failed.

## 3. Experimental Results and Analysis

In this section, we will analyze and compare the performance and overhead of the LA-FD detector through experiments. In order to make the results more convincing, detection processes have been designed according to the configuration of current mainstream mobile devices (ARM2440 processor, 400 MHz clock speed, 512 M RAM, 1,200 mAh battery capacity). The monitoring process is located in Harbin and connected to the Internet through WiFi. The monitored processes uses the configuration described in Section 2.3. There are two set of servers representing two typical network environments. one is located in Beijing (dataset 1) and the other is located in Pittsburgh (dataset 2). Experimental references are selected from several major implementations of accrual detectors such as Hayashibara's ϕ-failure detector (ϕ-FD) [11], Benjamin's new accrual detector (NAD) [15] and Avinash's improved ϕ-failure detector (I-ϕ-FD) [10]. All experiments are focused on two aspects of LA-FD: detection accuracy and system overhead.

### 3.1. Analysis of Detection Accuracy

**Figure 4.**
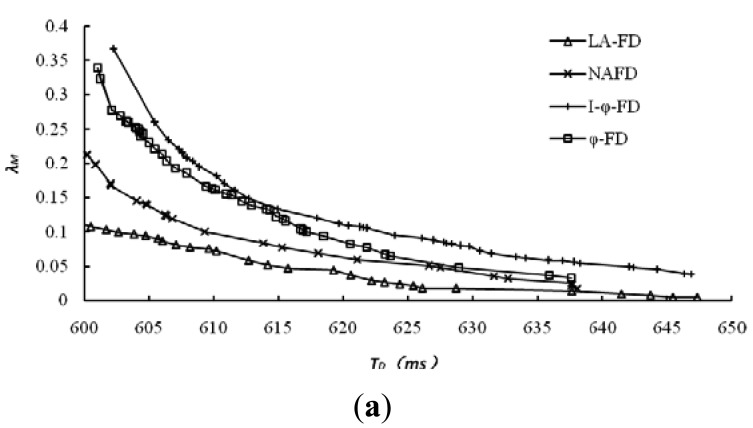

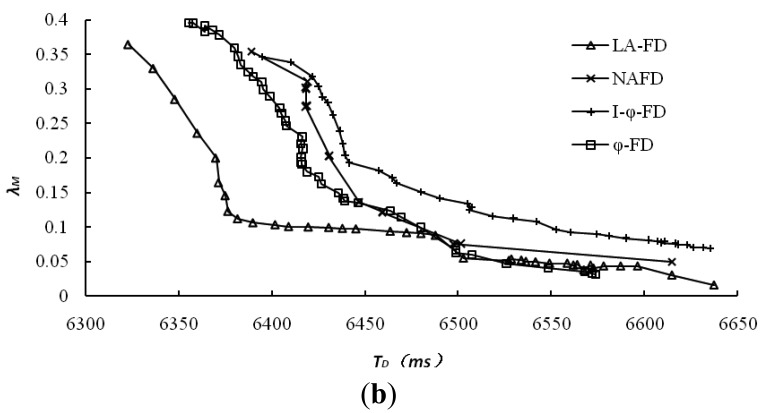
Average mistake rate and detection delay.

The accuracy of accrual failure detector is usually affected by two main factors. One is detection delay, and lower detection delay will reduce the accuracy of detection results; the other is the threshold set by upper applications for the suspicion level *sl*, and higher threshold leads to higher accuracy. However, in comparative experiments, different implementations of accrual failure detector use different approaches to calculate *sl* and its threshold. For the same detection data, we have collected all the detection results and related data from different detectors under multiple sets of thresholds. The relationship between the average mistake rate (*λ_M_*) and detection delay has been explored and the results are shown in Figure 4, where (a) and (b) represent the detection results for dataset 1 and dataset 2, respectively.

It's obviously from Figure 4 that LA-FD has demonstrated higher detection accuracy in both of the different network environments. Under the same accuracy requirement (mistake rate *λ_M_* in Y-axis), LA-FD has lower detection delay. This point is more obvious under poor network conditions (Figure 4(b)). I-ϕ-FD based on exponential distribution has the worst detection performance in both of the network environments. so, the assumption of exponential distribution is only suitable for the specific P2P systems and normal distribution can describe the message transmission delay more accurately.

### 3.2. Comparison of System Overhead

Since accrual failure detector needs to calculate detector parameters and maintain the history window of detection messages for each detection period, these two factors are the main reason for different system overhead in accrual detectors. A large history window will improve the prediction accuracy for model parameters and has a certain impact on the calculation accuracy for detector parameters. Meanwhile, the maintenance of history window will cause more overhead. For the experiments in this section, we have selected different historical window size settings and have made a detailed comparison of CPU utilization.

**Figure 5.**
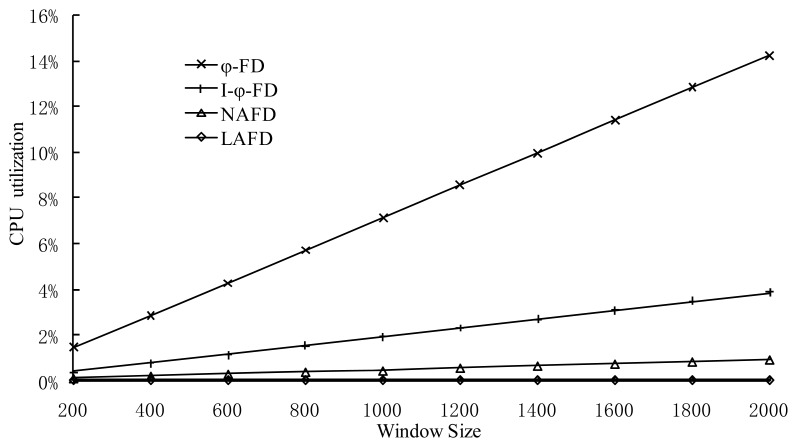
Comparison of CPU overhead.

It can be seen from the Figure 5 that the CPU overhead is the heaviest in the ϕ-detector based on normal distribution and it grows the fastest as the window size changes. This is because the workload for calculating parameters of the normal distribution model is the most, and every time it needs the statistical data from the entire window. The overhead of LA-FD is the least (about 0.08%), and it isn't affected by window size. Each process in the experiment shown in Figure 5 only maintains five connections. In large-scale P2P systems, in order to maintain a high locating efficiency, each process is generally required to maintain *logN* (*N* is the number of processes in the system) connections. Therefore, the fact that LA-FD can reduce CPU overhead is more significant in real systems.

## 4. Conclusions

Accrual failure detector can adapt to the changes in network conditions and on this basis, it can satisfy the different QoS requirements of multiple applications. The accrual failure detector is a fundamental component to ensure the efficiency and scalability of applications in large-scale distributed systems. Aiming at the characteristics that resources is constrained in mobile network equipment like cell phones and tablet PCs, LA-FD has been proposed as an accrual failure detector of class *◊P_ac_* [9] in this paper. It does not need the probability distribution for message transmission time and the maintenance costs for message history window. LA-FD can provide adaptive detection service to multiple applications with very low overhead. Experimental analysis has shown that compared to several other implementations of accrual detectors, LA-FD maintains a high detection accuracy while effectively reducing system overhead and it meets the needs of major distributed applications.
